# Neural correlates of anxious distress in depression: A neuroimaging study of reactivity to emotional faces and resting‐state functional connectivity

**DOI:** 10.1002/da.23264

**Published:** 2022-05-10

**Authors:** Laura Nawijn, Richard Dinga, Moji Aghajani, Marie‐José van Tol, Nic J. A. van der Wee, Andreas Wunder, Dick J. Veltman, Brenda W. H. J. Penninx

**Affiliations:** ^1^ Department of Psychiatry, Amsterdam Neuroscience, Amsterdam UMC, Location VUmc Vrije Universiteit Amsterdam Amsterdam The Netherlands; ^2^ Department of Psychiatry, Amsterdam Neuroscience, Amsterdam UMC, Location Academic Medical Center University of Amsterdam Amsterdam The Netherlands; ^3^ Donders Institute for Brain, Cognition and Behaviour Radboud University Nijmegen The Netherlands; ^4^ Section Forensic Family & Youth Care, Institute of Education and Child Studies Leiden University Leiden The Netherlands; ^5^ Department of Biomedical Sciences of Cells and Systems University Medical Center Groningen, Cognitive Neuroscience Center Groningen The Netherlands; ^6^ Department of Psychiatry Leiden University Medical Center Leiden The Netherlands; ^7^ Boehringer Ingelheim Pharma GmbH & Co. KG, Translational Medicine and Clinical Pharmacology Biberach an der Riss Germany

**Keywords:** amygdala, anxiety, anxious distress specifier, depression, neuroimaging, salience

## Abstract

**Background:**

Comorbid anxiety disorders and anxious distress are highly prevalent in major depressive disorder (MDD). The presence of the DSM‐5 anxious distress specifier (ADS) has been associated with worse treatment outcomes and chronic disease course. However, little is known about the neurobiological correlates of anxious distress in MDD.

**Methods:**

We probed the relation between the DSM‐5 ADS and task‐related reactivity to emotional faces, as well as resting‐state functional connectivity patterns of intrinsic salience and basal ganglia networks in unmedicated MDD patients with (MDD/ADS+, *N* = 24) and without ADS (MDD/ADS−, *N* = 48) and healthy controls (HC, *N* = 59). Both categorical and dimensional measures of ADS were investigated.

**Results:**

MDD/ADS+ patients had higher left amygdala responses to emotional faces compared to MDD/ADS− patients (*p* = .015)—part of a larger striato‐limbic cluster. MDD/ADS+ did not differ from MDD/ADS− or controls in resting‐state functional connectivity of the salience or basal ganglia networks.

**Conclusions:**

Current findings suggest that amygdala and striato‐limbic hyperactivity to emotional faces may be a neurobiological hallmark specific to MDD with anxious distress, relative to MDD without anxious distress. This may provide preliminary indications of the underlying mechanisms of anxious distress in depression, and underline the importance to account for heterogeneity in depression research.

## 1 INTRODUCTION

Anxiety and comorbid anxiety disorders are highly prevalent in individuals with major depressive disorder (MDD), with occurrence of lifetime anxiety disorders in MDD patients estimated at 46%–78% (Kessler et al., 2015; Lamers et al., 2011). Also in the absence of a formal anxiety disorder, high levels of anxiety are common in people with MDD (Ionescu, Niciu, Henter, et al., 2013). Therefore, in the DSM‐5 an anxious distress specifier (ADS) has been included for the diagnosis of MDD (American Psychiatric Association, 2013), capturing common core symptoms of anxious emotions and cognitions. The criteria for ADS are the presence of at least two of the following symptoms: (1) feeling keyed up or tense, (2) restlessness, (3) difficulty concentrating due to worry, (4) feeling that something awful may happen, and (5) feeling that one may lose control of oneself. Prevalence of ADS in MDD is estimated at 54%–75% (Gaspersz et al., 2017b; Hasin et al., 2018; Rosellini et al., 2018). While sharing symptoms with generalized anxiety disorder (GAD; feeling tense, restlessness, worry) and panic disorder (PD; fear of losing control of oneself), the ADS only partially overlaps with DSM‐IV and DSM‐5 comorbid anxiety disorders in depression (Cohen's *κ* =  0.09–0.21, agreement 35%–71%) (Gaspersz et al., 2017b; Rosellini et al., 2018; Zimmerman et al., 2019). For example, 23% of MDD patients with ADS were shown not to have any current anxiety disorder, and vice versa 51% of MDD patients without ADS did have a current anxiety disorder (Gaspersz et al., 2017b). The ADS also has been shown moderate associations with commonly used anxiety questionnaires (e.g., HAMD‐anxiety/somatization subscale, HAM‐A, BAI), suggesting these measure somewhat different constructs (Gaspersz et al., 2017b; Zimmerman et al., 2018). This partial discordance may be explained by the focus of ADS on anxious emotions and cognitions, while disregarding explicit physiological anxiety symptoms (e.g., chest pain, sweating, nausea), behavioral anxiety symptoms (e.g., avoidance), and specific fears, which are included in most anxiety scales and the diagnostic criteria for anxiety disorders (American Psychiatric Association, 2013; Penninx et al., 2021).

Notably, the DSM‐5 ADS outperformed the presence of DSM‐IV comorbid anxiety disorders in predicting MDD course and treatment outcomes (Gaspersz et al., 2017a, 2017b), self‐reported functional impairment, autonomic arousal, stress levels (Rosellini et al., 2018), and white matter structure (Heij et al., 2019), suggesting it captures a different and arguably clinically, functionally, and neurobiologically more relevant aspect of anxious depression. Considering these findings, and the relative ease with which the ADS can be administered (i.e., a five‐item checklist), the DSM‐5 ADS seems a relevant and easily accessible tool to identify patients at risk in an early stage.

Anxious depression may be a distinct subtype of depression, with not just clinically but also neurobiologically different profiles relative to nonanxious depression (Gaspersz et al., 2018). However, neurobiological research investigating anxious depression is sparse and heterogeneous (Ionescu, Niciu, Henter, et al., 2013; Zimmerman et al., 2019), and has focused mostly on MDD with comorbid anxiety disorders or dimensional measures of anxiety, whereas less studies thus far have investigated the ADS. Brain structures thought to be involved in anxious distress are the amygdala, hippocampus, striatum, anterior insula, and anterior cingulate cortex (ACC), which play a role in the processing of threatening and salient stimuli in the environment. The amygdala takes a central position here, acting as an integrative hub receiving input from areas involved in sensory processing, connected to areas involved in affective processing, and initiating arousal and stress responses (Dunsmoor & Paz, 2015; Roozendaal et al., 2009). These regions are strongly interconnected and form the key nodes of salience and limbic/basal ganglia networks (BGN; Laird et al., 2011; Seeley et al., 2007), displaying hyperactivity and altered connectivity in anxiety in depression (Crane et al., 2016; Fitzgerald et al., 2019; Li et al., 2015; C.‐H. Liu et al., 2015; Mehta et al., 2018; Oathes et al., 2015; Pannekoek et al., 2015; Price et al., 2017). A recent meta‐analysis of task‐related fMRI studies across mood and anxiety disorders indeed reported overlapping hyperactivation in amygdala and other salience‐processing areas during negative affective tasks in mood‐ and anxiety disorders relative to controls (Janiri et al., 2019). Furthermore, symptoms of anxious avoidance and threat dysregulation were consistently associated with amygdala–insula hypoconnectivity across depression and anxiety samples and controls, and behavioral threat responses to amygdala hyperreactivity to negative emotional faces (Goldstein‐Piekarski et al., 2021). Diverging neurobiological correlates of depression and anxiety have also been suggested, such as *decreased* limbic and striatal connectivity and reactivity in MDD without comorbid anxiety disorders or high levels of anxiety, and *increased* limbic and striatal connectivity and reactivity in MDD with comorbid anxiety disorders or high levels of anxiety (Oathes et al., 2015; Pannekoek et al., 2015), thought to reflect differential deficits in approach and avoidance behaviors in depression and anxiety (reviewed by Bruder et al., 2017; Williams, 2017). Taken together, increased salience processing of affective stimuli may form shared underlying neural phenotypes of anxiety and depression and may be present particularly in depression with high levels of anxiety (reviewed in Gaspersz et al., 2018; Ionescu, Niciu, Mathews, et al., 2013).

Possibly, more focused anxiety measures such as the ADS may have more specific distinguishing neurobiological features within MDD participants than the presence of any comorbid anxiety disorder. Indeed, compared to the presence of comorbid anxiety disorders, the ADS was better able to capture clinical outcome variables (Gaspersz et al., 2017a, 2017b), and differences in structural fronto‐limbic connectivity (Heij et al., 2019). The ADS, focusing on anxious emotions and cognitions, may be closer to specific neurobiological substrates of psychological anxiety, when compared to formal anxiety disorders or measures of anxiety based on combinations of psychological, behavioral, and physiological anxiety symptoms (Taschereau‐Dumouchel et al., 2022). Though clinical studies show promise for its clinical value, neurobiological studies investigating ADS thus far are very limited. A more clearly delineated neurobiological profile of anxious depression may inform us on the underlying mechanisms of the worse clinical prognosis observed related to the ADS, disentangle heterogeneity in MDD, and ultimately aid (tailoring of) its treatment (Ionescu, Niciu, Mathews, et al., 2013).

Thus, in the current study we investigated if the presence of the DSM‐5 ADS in MDD patients was associated with specific neural reactivity patterns relative to MDD participants without ADS and healthy controls. To assess specificity of the neural correlates of anxious distress relative to traditional DSM‐based diagnoses, we also compared MDD participants with and without comorbid anxiety disorders, and all MDD participants to controls. Based on previous literature indicating the amygdala as a key region involved in anxiety, we were specifically interested in amygdala responses to emotionally salient stimuli. To probe this, an emotional faces task was used, known to robustly elicit amygdala responses. Furthermore, based on network models of anxiety and previous findings in anxious depression implicating the interconnected salience and limbic/BGN, which include brain structures that are associated with amygdala functioning, such as the hippocampus, striatum, anterior insula, and ACC, we further investigated functional connectivity of these networks.

## 2 MATERIALS AND METHODS

### 2.1 Participants and procedures

Participants were recruited from the Netherlands Study of Depression and Anxiety (NESDA), a longitudinal observational cohort study (Penninx et al., 2008). Out of 2981 NESDA baseline respondents, 301 native Dutch‐speaking participants aged between 18 and 57 years participated in the neuroimaging study (Van Tol et al., 2010). Participants met the DSM‐IV criteria for a diagnosis of MDD and/or anxiety disorder (PD, social anxiety disorder [SAD], GAD) in the past 6 months, or no lifetime DSM‐IV diagnosis (control group). The exclusion criteria for the neuroimaging study were: (1) presence of lifetime axis‐I disorders other than MDD or anxiety disorders; (2) use of psychotropic medication; (3) major systemic and/or neurological disorders; (4) systolic blood pressure >180 mmHg and/or diastolic blood pressure >120 mmHg; (5) past year alcohol and/or drugs abuse or dependency; (6) general MRI contraindications. For the current study we additionally excluded anxiety disorder patients without MDD, and MDD participants using antidepressant medication, to limit potentially confounding effects of psychostimulants, leaving a sample of healthy control participants (*n* = 68) and unmedicated participants with MDD (*n* = 102). Imaging sessions were performed at the university medical centers in Amsterdam, Leiden, and Groningen. This study was approved by the Ethical Review Boards of each participating center. After receiving written information, each participant gave written informed consent.

### 2.2 MEASURES

#### 2.2.1 Mood and anxiety

All participants were interviewed with the Composite International Diagnostic Interview (CIDI; Robins et al., 1988) version 2.1 to establish the presence of depressive and anxiety disorders, according to the Diagnostic and Statistical manual of Mental Disorders fourth edition (DSM‐IV), administered by trained interviewers. Age of depression onset and recurrency of MDD (i.e., presence of a single MDD episode vs. multiple episodes) were assessed as part of the clinical interview.

The ADS was constructed by five self‐reported items from the Inventory of Depressive Symptomatology (IDS; Rush et al., 1996) and the Beck Anxiety Inventory (BAI; Beck et al., 1988), administered at the day of scanning, that matched directly with the five criteria for the DSM‐5 ADS (Gaspersz et al., 2017b; Heij et al., 2019). According to the DSM‐5, the ADS was present (ADS+) when a participant endorsed ≥2 of the following symptoms (i.e., scoring ≥2 on a 0–3 scale): (1) feeling keyed up or tense; (2) feeling unusually restless; (3) difficulty concentrating because of worry; (4) fear that something awful might happen; (5) feeling you might lose control of oneself. MDD patients were thus divided into two groups; those with and without ADS (MDD/ADS+, MDD/ADS−). Furthermore, an ADS dimensional score was determined by adding up the scores of the five IDS/BAI items (range 0–15). To assess overlap with comorbid anxiety disorders, we likewise distinguished MDD participants with and without current comorbid anxiety disorder (MDD/ANX+, MDD/ANX−) (i.e., PD, SAD, GAD). As additional measures of anxiety, the Fear questionnaire (Marks & Mathews, 1979) and Penn State Worry questionnaire (Meyer et al., 1990) were administered. Functional disability in the past 30 days was assessed using the World Health Organization Disability Assessment Schedule (WHO DAS II; Chwastiak & Von Korff, 2003).

#### 2.2.2 Imaging paradigms, acquisition, and preprocessing

The emotional faces paradigm was based on the event‐related emotional paradigm used by Demenescu et al. (2011). Angry, fearful, sad, happy, and neutral facial expressions were presented one at a time (stimulus duration 2.5 s) and participants were instructed to indicate the face's gender with button presses. The control condition consists of scrambled faces with a motor control task. For more details see Supporting Information Methods A1. The resting state fMRI scan lasted 7 min and 40 s. Resting state data were acquired in the darkened MR room, participants were instructed to lie still with their eyes closed and to not fall asleep (Veer et al., 2010). Compliance to these instructions was verified as part of the exit interview. Participants were scanned in Philips 3‐Tesla MRI scanners, equipped with either a SENSE‐6 (AMC) or a SENSE‐8 channel head coil (LUMC, UMCG). For scan parameters and preprocessing see Supporting Information Methods A2.

### 2.3 STATISTICAL ANALYSES

First, demographic, clinical, mood, and anxiety measures were inspected for normality and outliers. To investigate group differences (MDD/ADS− vs. MDD/ADS+; HC vs. MDD) in these measures, independent sample *t*‐tests, *χ* ^2^ tests, and Mann–Whitney *U *tests were performed depending on variable distribution. IBM SPSS statistics version 24 was used for demographic and clinical analyses.

#### 2.3.1 Emotional faces task analyses

For the emotional faces task, each participant's imaging data were analyzed in the context of the General Linear Model, using delta functions to model responses to stimuli (happy, angry, sad, fearful, neutral, scrambled faces). First‐level contrast images for emotional faces (happy, angry, sad, fearful) versus scrambled faces were produced. Group analyses were performed using the FEAT toolbox of FSL. First‐level contrasts were entered into second‐level analyses of covariance (ANCOVA) for group comparisons, including group as a three‐level factor (MDD/ADS+, MDD/ADS−, Control). As we were primary interested in group differences between MDD/ADS+ and MDD/ADS−, a priori*‐*defined simple contrasts were performed in FSL FEAT, using FLAME 1 mixed‐effects modeling (recommended for estimation of higher‐level activation in FSL FEAT [Woolrich et al., 2004] and showing robust performance [Eklund et al., 2016]), correcting for covariates (age, sex, education, scan‐site). To correct for multiple comparisons across voxels, test statistics were thresholded using cluster‐based thresholding (z = 2.3) based on Gaussian Random Field Theory as incorporated in FSL FEAT (Woolrich et al., 2004), and considered significant at a corrected cluster probability threshold of *p* < .05. Significant group differences were followed up with additional comparisons between MDD/ADS+ versus controls, and MDD/ADS− versus controls to test deviations from the control reference group. Additionally, second‐level regression analyses were run, investigating associations between the dimensional ADS score and reactivity to emotional faces in all participants. As secondary analyses, to assess the specificity of the neural correlates of ADS relative to traditional DSM‐based diagnoses, we similarly compared MDD/ANX+ and MDD/ANX−; and all MDD participants taken together irrespective of anxiety (MDD_all_) relative to controls in separate ANCOVA's.

We had a primary focus on amygdala responsiveness, employing region of interest (ROI) analyses based on Harvard‐Oxford anatomical templates for the left and right amygdala in one bilateral amygdala mask (no probability threshold for inclusive amygdala assessment). For sake of completeness, we additionally ran all group analyses at whole‐brain level. To illustrate significant associations and post hoc sensitivity analyses, mean percent signal change values were extracted from a 5 mm sphere around the peak‐voxels using FEATquery.

#### 2.3.2 | Resting state functional connectivity analyses

For the resting‐state scan, we investigated large‐scale intrinsic functional connectivity networks. Standard group independent component analyses (ICA) were carried out using probabilistic ICA in FSL MELODIC (Beckmann et al., 2005), separating the four‐dimensional functional data into 20 independent components. Our primary interest was in the salience network (SN) and the basal ganglia/limbic network (BGN), identified from the 20 components based on visual inspection and cross‐correlations with intrinsic connectivity network templates by Laird et al (2011).

Next, the subject‐specific component maps were identified with dual regression in FSL (Nickerson et al., 2017). Subject‐specific component maps of interest were subsequently entered in second‐level between‐group analyses using FSL's Randomise permutation‐testing tool. ANCOVA's including group as a three‐level factor (MDD/ADS+, MDD/ADS−, Control) were used to determine group differences in intrinsic functional connectivity networks. Our primary analyses focused on a priori*‐*defined simple‐contrast group differences between MDD/ADS+ and MDD/ADS− while correcting for covariates (sex, age, education, scan site), performed with Randomise. To correct for multiple comparisons across voxels, test statistics were corrected using family‐wise error and threshold‐free cluster enhancement implemented in Randomise. Significant group differences were followed up with additional comparisons between MDD/ADS+versus controls, and MDD/ADS− versus controls to test deviations from the control reference group. Additionally, second‐level regression analyses were run, investigating associations between the dimensional ADS score and functional connectivity patterns in all participants. Furthermore, as secondary analyses, to assess specificity of the neural correlates of the ADS relative to traditional DSM‐based diagnoses, we additionally compared MDD/ANX+, MDD/ANX−, and controls; and all MDD participants were taken together irrespective of anxiety (MDD_all_) relative to controls.

As two networks of interest were tested (SN, BGN), *p *values were additionally corrected for multiple comparisons across networks using the false discovery rate (FDR) method. To illustrate significant associations and to run post hoc sensitivity analyses, mean percent signal change values were extracted from a 5‐mm sphere around the peak voxels.

## 3 RESULTS

### 3.1 Sample characteristics

For two control‐ and two MDD participants the ADS could not be calculated due to missing IDS or BAI items, another 7 controls and 23 MDD participants did not have any imaging data of sufficient quality (e.g., due to excessive movement, signal quality, registration problems, see Sections 3.2 and 3.3), leaving an overall imaging sample of *n* = 136 (controls: *n* = 59, MDD: *n* = 77). In total, 36.4% of MDD participants (*n* = 28) scored positive on the ADS. Average scores on the ADS dimensional measure were significantly higher in MDD patients (*M* = 4.51, SD = 3.48) compared to healthy controls (*M* = 0.46, SD = 1.02; *p* < .001), and in MDD/ADS+ patients (*M* = 8.14, SD = 2.37) compared to MDD/ADS− patients (*M* = 2.43, SD = 1.97; *p* < .001, see Table 1), inherent to the definition of the ADS groups. Within the imaging sample, the ADS showed moderate overlap with diagnosis of current comorbid anxiety disorders; Of the MDD participants with ADS, 64.3% also met the criteria for one or more anxiety disorders (SAD/GAD/PD according to DSM‐IV), whereas of the MDD participants without ADS, 63.3% did not meet criteria for any of the anxiety disorders (Cohen's *κ *= 0.096, agreement 63.6%, Table 1). MDD/ADS+ participants had significantly more often a current anxiety disorder diagnosis compared to MDD/ADS− (64.3% vs. 36.7%, *p* = .020), and a higher average number of anxiety disorders (*p* = .019). Particularly the presence of SAD was higher in MDD/ADS+ relative to MDD/ADS− (*p* = .038), whereas GAD (*p* = .299) and PD and/or agoraphobia (*p* = .141) did not show significant differences in prevalence. Compared to controls, MDD participants (MDD_all_) were significantly younger and less educated (*p* < .05; Table 1). MDD/ADS+ and MDD/ADS− did not differ significantly in any demographic characteristics, though MDD/ADS+ scored higher on several clinical characteristics compared to MDD/ADS−, such as higher IDS and BAI scores (Table 1), suggesting not only more severe anxiety symptoms but also more severe depressive symptoms. This difference remained when ADS items were excluded from the IDS, *t*(75) = −5.735, *p* < .001), and BAI total scores, *t*(75) = −5.796, *p* < .001). MDD/ADS+ participants further displayed significantly higher scores relative to MDD/ADS− on fear and worry symptoms (resp. *p* = .019, *p* = .002, Table 1). No significant differences were observed between MDD/ADS+ and MDD/ADS− in the age of MDD onset, MDD recurrence, or functional disability.

**Table 1:** Sample characteristics (*n* = 136).

	HC (*n* = 59)	MDD/ADS− (*n* = 49)	MDD/ADS + (*n* = 28)	HC versus MDD_ALL_		MDD/ADS+ versus MDD/ADS−	
*Demographic characteristics*							
Females, *n* (%)	37 (62.7%)	34 (69.4%)	19 (67.9%)	*χ* ^2^(1) = 0.559	*p* = .455	*χ* ^2^(1) = 0.019	*p* = .889
Age in years, mean (SD)	39.85 (9.49)	35.67 (10.59)	36.64 (11.66)	*t*(134) = 2.19	*p* = .034*	*t*(75) = −0.373	*p* = .711
Education in years, *n* (%)	14.44 (2.79)	12.45 (2.68)	11.82 (2.68)	*t*(134) = 4.707	*p* < .001***	*t*(75) = 0.989	*p* = .326
Current smokers, *n* (%)	11 (18.6%)	12 (24.5%)	12 (42.9%)	*χ* ^2^(1) = 2.742	*p* = .098	*χ* ^2^(1) = 2.802	*p* = .094
Site (Ams/Leid/Gron), *n* (%)	20, 26, 13	10, 22, 17	5, 10, 13	*χ* ^2^(2) = 5.775	*p* = .056	*χ* ^2^(2) = 1.051	*p* = .591
	(33.9%, 44.1%, 22.0%)	(20.4%, 44.9%, 34.7%)	(17.9%, 35.7%, 46.4%)				
*Mood & anxiety characteristics*							
Age of onset depression in years, mean (SD)	‐	22.88 (9.70)	24.54 (12.08)	‐		*t* (75) = −0.262	*p* = .397
Recurrent MDD, n (%)	‐	33 (67%)	15 (54%)	‐		*χ* ^2^(1) = 1.440	*p* = .230
Functional disability (WHODAS II score), mean (SD)	4.55 (5.62)	31.10 (14.43)	35.64 (11.62)	*t*(132) = −14.84	*p* < .001***	*t*(74) = −1.402	*p* = .083
Current anxiety diagnosis, *n* (%)	0 (0%)	18 (36.7%)	18 (64.3%)	‐		*χ* ^2^(1) = 5.433	*p* = .020*
Generalized anxiety disorder, *n* (%)	0 (0%)	9 (18.4%)	8 (28.6%)	‐		χ^2^(1) = 1.064	*p* = .299
Panic disorder and/or agoraphobia, *n* (%)	0 (0%)	13 (26.5%)	12 (42.9%)	‐		*χ* ^2^(1) = 2.166	*p* = .141
Social anxiety disorder, *n* (%)	0 (0%)	5 (10.2%)	8 (28.6%)	‐		*χ* ^2^(1) = 4.284	*p* = .038*
Number of current anxiety diagnoses, median (IQR)	0 (0.00‐0.00)	0 (0.00‐1.00)	1 (0.00‐2.00)	‐		*U* = 484.500	*p* = .019*
Lifetime anxiety diagnosis, *n* (%)	0 (0%)	27 (55.1%)	20 (71.4%)	‐		*χ* ^2^(1) = 1.997	*p* = .158
Anxious distress dimensional score, mean (SD)	0.46 (1.02)	2.43 (1.97)	8.14 (2.37)	*t*(134) = −8.654	*p* < .001***	*t*(74) = −11.372	*p* < .001***
Fear questionnaire, mean (SD)	9.24 (7.68)	19.29 (11.80)	38.14 (18.87)	*t*(132) = −6.971	*p* < .001***	*t*(74) = −5.364	*p* = .019*
Worry questionnaire, mean (SD)	18.48 (6.19)	34.87 (12.73)	43.09 (6.28)	*t*(124) = −11.068	*p* < .001***	*t*(68) = −2.918	*p* = .002**
Beck Anxiety Inventory score, mean (SD)	2.24 (2.69)	8.10 (6.81)	18.68 (9.35)	*t*(134) = −7.987	*p* < .001***	*t*(75) = −6.444	*p* < .001***
Depression severity (IDS score), mean (SD)	4.19 (4.20)	17.20 (10.03)	32.46 (9.17)	*t*(134) = −11.214	*p* < .001***	*t*(75) = −6.620	*p* < .001***
IDS mood/cognition subscale, mean (SD)	0.76 (1.38)	5.94 (3.72)	10.43 (2.70)	*t*(134) = −12.484	*p* < .001***	*t*(75) = −5.591	*p* < .001***
IDS anxiety/arousal subscale, mean (SD)	1.27 (1.42)	3.51 (2.28)	6.29 (1.72)	*t*(134) = −8.986	*p* < .001***	*t*(75) = −5.586	*p* < .001***

*Note*: Group comparisons: independent sample *t*‐tests (*t*) were used for continuous variables, *χ* ^2^ for categorical variables, Mann–Whitney *U* tests (*U*) for ordinal variables.

Abbreviations: HC, healthy controls; IDS, inventory of depressive symptomatology; IQR, interquartile range; MDD, major depressive disorder; MDD/ADS−, participants with major depressive disorder without anxious distress specifier; MDD/ADS+, participants with major depressive disorder and anxious distress specifier; MDDALL, all participants with major depressive disorder irrespective of anxious distress; WHODAS II, World Health Organisation Disability Assessment Schedule.

* *p* < .05;

** *p* < .01;

*** *p* < .001.

### 3.2 EMOTIONAL FACES TASK

Complete emotional faces task data were available for 83 unmedicated MDD participants and 59 controls. After preprocessing, eight participants were excluded due to excessive movement (>|2/5|mm or >|0.4|rad) and six to insufficient data quality (e.g., signal quality, registration problems). A final sample of 128 participants was available, including 24 MDD/ADS+ participants, 48 MDD/ADS− participants, and 55 controls. The main task effect (emotional faces > scrambled faces) yielded significant BOLD responses in the bilateral amygdala, hippocampus, fusiform gyrus, right inferior frontal gyrus, and other regions (Table S1 and Figure S1), comparable to previous meta‐analytic findings (Fusar‐Poli et al., 2009; Sabatinelli et al., 2011).

#### 3.2.1 Emotional faces reactivity in anxious distress

**Figure 1:**
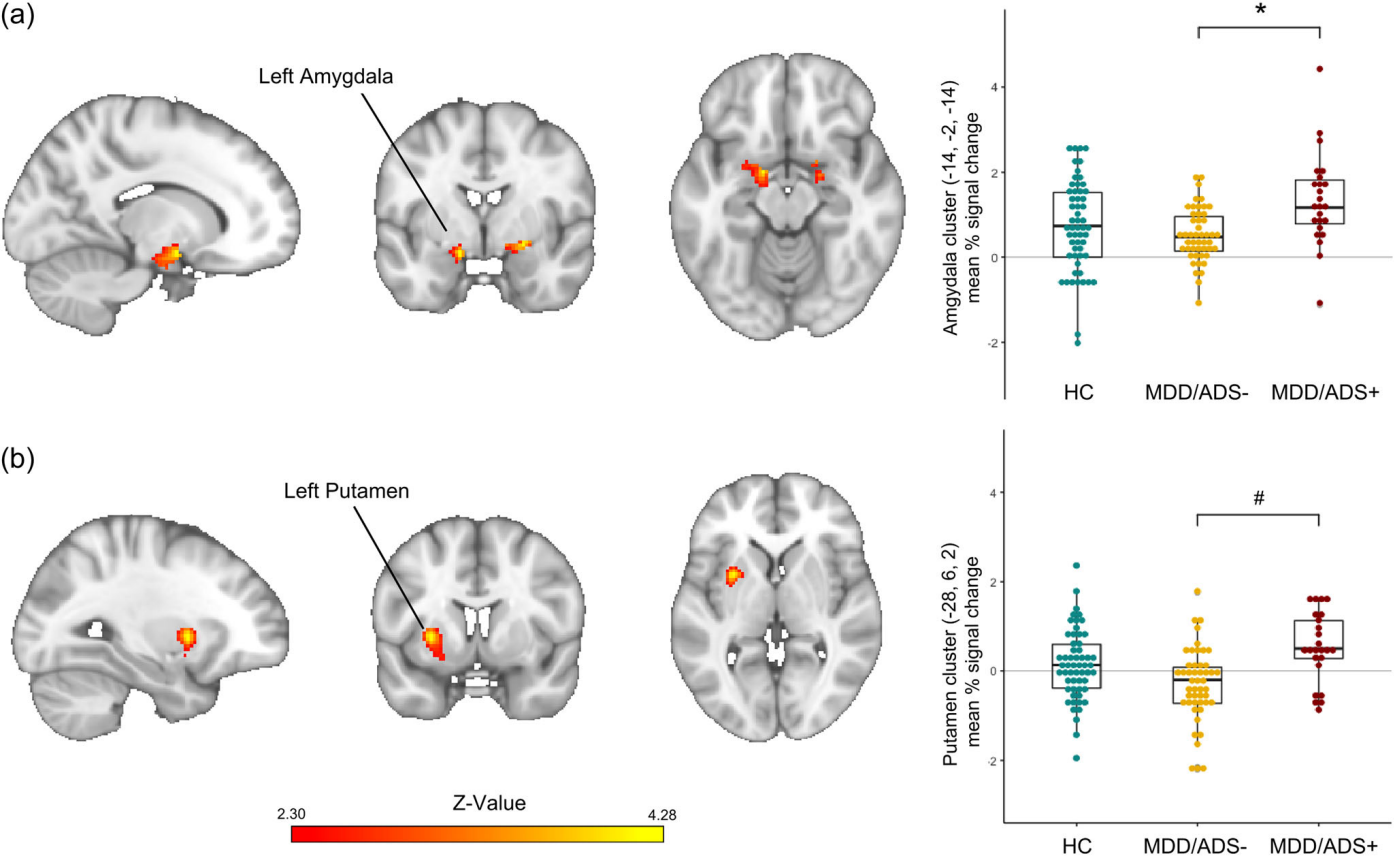
Increased amygdala and putamen responses to emotional faces in MDD with anxious distress. (a)Within amygdala region‐of‐interest analysis MDD patients with anxious distress (MDD/ADS+) show stronger left amygdala responses to emotional faces relative to MDD without anxious distress (MDD/ADS−) (MNI coordinates *x*, *y*, z −14, −2, −14, *Z* = 4.02, *k* = 167, *p* = 0.015), and stronger right amygdala responses relative to healthy controls (HC) (*x*, *y*, *z* 24, −2, −10; *Z* = 3.01, *k* = 79, *p* = 0.072), and boxplots showing left amygdala‐peak responses (extracted from 5 mm sphere around peak voxel at *x*, *y*, *z* −14, −2, −14) in HC, MDD/ADS−, and MDD/ADS+. (b) Within whole‐brain analysis, MDD/ADS+ show stronger responses to emotional faces in a cluster spanning the left putamen, amygdala, and hippocampus relative to MDD/ADS− (MNI‐coordinates *x*, *y*, *z* −28, 6, 2, *Z* = 4.28, *k* = 372, *p* = 0.052), and boxplot with left putamen‐peak responses (extracted from 5 mm sphere around peak voxel) in HC, MDD/ADS−, and MDD/ADS+. Heatmaps correspond to *Z*‐values, threshold *Z *≥ 2.30 (panel (a) masked to show only amygdala ROI), and are overlaid on an MNI standard brain template, right hemisphere in image corresponds to right hemisphere of the brain. Abbreviations: HC, healthy control; MDD/ADS−, major depressive disorder without anxious distress specifier; MDD/ADS+, major depressive disorder with anxious distress specifier. *p < .050 based on amygdala region‐of‐interest FSL FEAT MDD/ADS+ vs MDD/ADS− comparison, ^#^​​​​*p* < .100, based on whole‐brain FSL FEAT MDD/ADS+ vs. MDD/ADS− comparison.

In the primary comparisons within the bilateral amygdala ROI, MDD/ADS+ participants displayed stronger left amygdala responses towards emotional faces compared to MDD/ADS− (Figure 1a, *xyz* −14, −2, −14, *Z* = 4.02, *k* = 167, *p* = .015) and nonsignificantly in the right amygdala compared to controls (Figure 1a, *xyz* 24, −2, −10; *Z* = 3.01, *k* = 79, *p* = .072). There were no differences in amygdala reactivity in MDD/ADS− relative to controls. Whole‐brain analyses suggested this amygdala effect was part of a larger cluster spanning the left putamen, amygdala, and hippocampus, in which MDD/ADS+ displayed stronger responses relative to MDD/ADS− (Figure 1b, *xyz* −28, 6, 2; *Z* = 4.28, *k* = 372, *p* = .052), though this did not reach significance at the whole ‐brain level. MDD/ADS+ nor MDD/ADS− showed significant differences compared to controls in this cluster.

Post hoc analyses did not reveal significant group by emotion effects in amygdala hyperreactivity in MDD/ADS+ relative to MDD/ADS−. Although not all contrasts passed the significance threshold of *p* < .05, increased amygdala responses in MDD/ADS+ were present to similar extents in response to all types of negative and positive emotional faces included in the current contrast (i.e., happy, angry, sad, fearful), particularly in the left amygdala (see Table S2).

Sensitivity analyses run on mean percent signal change extracted from the left amygdala and putamen clusters for all participants suggest the differences in amygdala and putamen responses between MDD/ADS+ and MDD/ADS− were not driven by comorbid anxiety disorders or depression severity, as group differences remained significant after excluding participants with comorbid anxiety disorders and when correcting for the presence of comorbid anxiety disorders and depression symptom severity (i.e., IDS total scores, see Supporting Information  Results B1).

#### 3.2.2 Emotional faces reactivity in MDD and comorbid anxiety disorders

In secondary analyses, to assess DSM‐diagnosis‐based group differences in emotional faces reactivity, we further compared MDD/ANX+, MDD/ANX−, and controls, and MDD_all_ with controls. No differences in neural responses within the amygdala or other brain regions were observed when comparing MDD/ANX+ participants and MDD/ANX−. No significant differences in amygdala responses were observed between MDD_all_ and controls. In whole‐brain analyses, MDD_all_ displayed decreased lingual gyrus/precuneus responses to emotional faces relative to controls (Figure S2, *xyz* −20, −60, 2, *Z* = 4.40, *k* = 405, *p* = .035).

### 3.3 RESTING STATE FUNCTIONAL CONNECTIVITY

Complete resting state data were available for 79 unmedicated MDD participants and 58 controls. After preprocessing, nine participants were excluded due to excessive movement (>|2/5|mm or >|0.4|rad in any direction) and three to insufficient data quality (e.g., signal quality, registration problems). A final sample of 125 participants was available, including 27 MDD/ADS+ participants, 41 MDD/ADS− participants, and 57 controls. From ICA at the whole‐ group level the SN and BGN were identified (Figure S3); The SN comprised clusters in the bilateral anterior insula (AI) and the dorsal ACC (dACC); the BGN comprised clusters in the striatum, thalamus, and amygdala, showing clear visual overlap and significant cross‐correlations with corresponding templates by Laird et al. (2011) (SN: *r* = .31, BG: *r* = .50).

#### 3.3.1 Resting state functional connectivity in anxious distress

**Figure 2:**
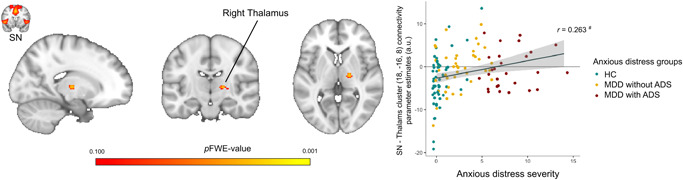
Subthreshold positive correlation between anxious distress specifier dimensional scores and salience network functional connectivity. Across MDD patients and controls (*n* = 125) anxious distress specifier (ADS) dimensional scores were subthreshold positively correlated with salience network (SN) (coronal view of the SN shown in small image top left) connectivity in the right thalamus (*xyz* 18 −16 4, *p* = 0.026, *p*_FDR_ = 0.052), shown in the middle image in sagittal, coronal, and horizontal slice. On the right, a scatterplot with extracted mean parameter estimates from a 5 mm sphere around the peak voxel, including a linear regression line (*r* = 0.263) and 95% confidence intervals to illustrate the correlation. Heatmaps correspond to *p *values, threshold *p* < 0.100, and are overlaid on an MNI standard brain template. Right hemisphere in image corresponds to right hemisphere of the brain. ADS, anxious distress specifier; a.u., arbitrary units; HC, healthy control; MDD, major depressive disorder; SN, salience network. ^#^ *p* < 0.050 based on whole‐brain neuroimaging regression analyses, but not significant after FDR correction.

In our primary ADS‐based group comparisons, no significant differences were observed between MDD/ADS+, MDD/ADS−, and controls, in functional connectivity of the SN or BGN. Across all participants (*n* = 125), ADS dimensional scores were positively associated with SN‐thalamus functional connectivity, though this did not hold after FDR correction for the multiple networks of interest (Figure 2, *xyz* 18 −16 4, *k* = 37, *p* = .026, *p*_FDR_ = 0.052). Also, post hoc sensitivity analyses showed this association was no longer significant when correcting for depression severity or depression status, nor when including only MDD patients (*n* = 68). ADS dimensional scores were not significantly associated with BGN FC.

#### 3.3.2 Resting state functional connectivity in MDD and comorbid anxiety disorders

As secondary analyses, to assess DSM‐diagnosis‐based group differences in functional connectivity, we further compared MDD/ANX+, MDD/ANX− and controls, and MDD_all_ with controls. No significant functional connectivity differences were observed between MDD/ANX+, MDD/ANX−, and controls in SN FC. Nonsignificant increased BGN‐middle frontal gyrus connectivity was observed in MDD/ANX+ relative to MDD/ANX− (*xyz *−36 30 36, *k* = 20, *p* = .056, *p*_FDR_ = 0.112). MDD_all_ was associated with increased SN‐putamen FC relative to controls though this was no longer significant after FDR correction for including two networks of interest (*xyz* 28 −18 2, *k* = 45, *p* = .026, *p*_FDR_ = 0.052; Figure S4). There were no significant differences in BGN FC between MDD_all_ and controls.

## 4 DISCUSSION

In the current study, we investigated if the presence of the DSM‐5 ADS in a sample of unmedicated MDD participants was associated with specific neural reactivity to emotional faces, and functional connectivity patterns during rest. We observed increased amygdala reactivity—part of a larger striato‐limbic cluster—to emotional faces in MDD with ADS (MDD/ADS+), relative to MDD without ADS (MDD/ADS−). There was also a suggestion of higher amygdala reactivity in MDD/ADS+ relative to controls, but this did not reach statistical significance (*p* = .072). There was no indication of differences in amygdala reactivity in MDD/ADS− relative to controls.

The MDD‐ADS groups did not differ significantly in resting‐state functional connectivity of the salience (SN) or BGN. ADS dimensional scores were subthreshold positively associated with SN‐right thalamus functional connectivity, warranting further investigation. However, subthreshold higher SN‐right putamen connectivity was also observed when comparing all MDD patients (MDD_all_) to controls, suggesting SN hyperconnectivity may not be specific to ADS but rather related to depression in general.

### 4.1 Anxious distress associated with amygdala & striatal hyperreactivity to emotional faces

The observed hyperreactivity of the amygdala in anxious distress, part of a cluster also spanning the putamen and hippocampus, fits with previous meta‐analytic observations of limbic and striatal hyperresponsiveness as overlapping constructs in depression and anxiety (Janiri et al., 2019). Interestingly, hyperresponsiveness was observed to a similar extent across emotional expression (happy, angry, sad, fearful), suggesting this may be independent of valence and affective context (e.g., present across threatening, sad, or positive social situations). Increased responsiveness in limbic areas to emotional stimuli may lead to enhanced biological stress responses involving the amygdala, through stimulation of the hypothalamic–pituitary–adrenal axis and sympathetic nervous system (Gold, 2015), potentially underlying higher levels of subjective arousal and perceived stress that have been associated with the ADS (Rosellini et al., 2018). The striatum is usually implied in goal‐directed behavior towards positive or rewarding stimuli, and also plays a role in processing of negative stimuli and punishment (X. Liu et al., 2011). Hyperreactivity of the striatum may be involved in negative bias or dysfunctional valence assessment of emotional faces in MDD (Lai, 2014).

Amygdala and putamen hyperresponsivity in MDD/ADS+ could not be explained by comorbid anxiety disorders or depression severity, as group differences remained significant when controlling for depression severity and controlling for or excluding comorbid anxiety disorders. Furthermore, no significant differences were observed in limbic or striatal reactivity when comparing MDD patients with (MDD/ANX+) and without (MDD/ANX−) comorbid anxiety disorders, nor when comparing MDD_all_ to controls. Though within our sample it was not feasible to formally compare the ADS with comorbid anxiety disorders, the current findings may cautiously suggest that the ADS captures a specific neurobiological aspect of anxious depression that is not picked up by diagnosing comorbid anxiety disorders. Based on previously discussed differences between DSM‐based anxiety disorders and the ADS, that is, the former including combinations of psychological, behavioral, and physiological symptoms of anxiety and specific fears, and the ADS focusing more on psychological anxiety symptoms, the amygdala and limbic‐striatal hyperreactivity may be specific to psychological anxiety symptoms as assessed with the ADS. Also, heightened amygdala/limbic‐striatal hyperactivity may not be (equally) present across the different anxiety disorders included in this study. Dimensional anxiety levels have previously shown stronger associations with functional neuroimaging measures in MDD than discrete (comorbid) anxiety disorders (Fitzgerald et al., 2019; Oathes et al., 2015). Similarly, anxiety symptom severity but not anxiety disorder diagnosis explained variance in amygdala connectivity during emotional faces in a sample of PD and SAD patients (Demenescu et al., 2013). Potentially, there are also differences *between* anxiety disorders, which may dilute neurobiological substrates of anxious depression when grouped together based on different comorbid anxiety disorders. Indeed, other studies have suggested anxiety disorders may present a heterogeneous group of disorders with different clinical and pathophysiological mechanisms (Morneau‐Vaillancourt et al., 2020). On the other hand, though not completely overlapping, we must acknowledge the overlap between ADS, anxiety disorders, and other dimensional anxiety and depression measures, and realize these concepts are inherently intertwined (Penninx et al., 2021; ter Meulen et al., 2021). Together, these findings may suggest that psychological anxiety symptoms, such as those assessed with the ADS, may track different neurobiological and clinical constructs within MDD than diagnosis of comorbid anxiety disorders.

Of note, we did not observe significant differences in amygdala reactivity between MDD/ADS+ and healthy controls (*p* = .072), although the difference in amygdala reactivity between MDD/ADS+ and healthy controls was in the same direction as the difference between MDD/ADS+ and MDD/ADS−. Alternatively, if the amygdala hyperreactivity observed in MDD/ADS+ is only present relative to MDD/ADS− but not healthy controls, this may imply that MDD/ADS− display amygdala *hypo‐*reactivity relative to controls, thereby indicating *opposing* amygdala responses in anxious and non‐anxious MDD as has been suggested by, for example, Oathes et al. (2015). Although the current data do not support hypo‐reactivity in MDD/ADS−, future research is needed to confirm if MDD/ADS+ indeed display amygdala hyperreactivity relative to both MDD/ADS− and healthy controls, while MDD/ADS− show similar amygdala activity as controls.

Overall, our findings further emphasize the need to address heterogeneity in depression, as subgroups and symptom dimensions may differ in underlying neurobiology (Fu et al., 2019), making it crucial to progress in treatment and research of depression (Fried, 2017). Heterogeneity within patient groups may also explain previous inconsistent findings with respect to limbic and striatal responses in neuroimaging meta‐analyses of MDD, with both increased, decreased, and no differences in amygdala and putamen responses to emotional stimuli being reported in MDD relative to controls (Groenewold et al., 2013; Lai, 2014; Müller et al., 2017; Palmer et al., 2015). Though not part of our primary research questions, decreased lingual gyrus/precuneus responses to emotional faces were observed in MDD_all_ relative to controls. This builds upon previous literature indicating the relevance of the precuneus in MDD, as the key node of the default mode network (Gong et al., 2020; Kaiser et al., 2015) potentially involved in rumination (Hamilton et al., 2015); and the lingual gyrus, which aids in visual processing of emotional faces (Fusar‐Poli et al., 2009), and may play a role in biased processing of emotional stimuli in MDD (Gong et al., 2020).

### 4.2 Anxious distress not convincingly or specifically related to resting‐state functional connectivity

We observed no differences between ADS groups in functional connectivity of the SN or BGN. ADS dimensional scores were positively associated with SN–thalamus connectivity. However, the association was no longer significant after correction for multiple testing, nor when correcting for depression severity or depression status. Fitting with this notion, MDD_all_ was associated with subthreshold SN–putamen hyperconnectivity relative to controls, in a region proximal to the ADS‐related thalamus cluster, further suggesting the observed subthreshold SN hyperconnectivity may be driven by depression (severity) rather than anxious distress.

Alterations in SN connectivity have previously been reported in MDD (meta‐analysis/reviews by Dutta et al., 2014; Kaiser et al., 2015) but also specifically in anxious depression (Mehta et al., 2018; Oathes et al., 2015; Pannekoek et al., 2015; Price et al., 2017). The SN and regions like the putamen and thalamus form a well‐connected circuit, integrating sensory information, guiding attention towards motivationally relevant stimuli, and enabling goal‐directed behavior, that may be central to a broad range of psychiatric symptoms (Peters et al., 2016). Interestingly, a large recent study suggested SN alterations were associated most consistently with anxious arousal symptoms, but were also related to anhedonia, negative bias, threat dysregulation, and cognitive dyscontrol, suggesting the SN may play a broader role across depression and anxiety symptoms (Goldstein‐Piekarski et al., 2021). Of relevance, it is difficult to tease apart anxious depression from depression severity as the two often go hand in hand (e.g., Gaspersz et al., 2017b), as was also the case in the current sample. Thus, future research is needed to disentangle if SN dysfunction is specific to anxious depression or a general hallmark of depression status or depression severity.

### 4.3 | Limitations and strengths

To our knowledge, this is one of the first studies investigating functional neuroimaging correlates of the DSM‐5 ADS in MDD. We included a medication‐free, and clinically well‐phenotyped group of MDD participants. This allowed us to not only compare MDD with and without anxious distress, but to also run sensitivity analyses investigating MDD with and without comorbid anxiety. The relatively small sample size however, and the smaller proportion of MDD participants with ADS compared to previous studies (36% vs. 54%–75%; Gaspersz et al., 2017b; Hasin et al., 2018; Rosellini et al., 2018), may have limited the power to detect small effect sizes, and did not allow us to formally compare different measures of anxious depression or different types of comorbid anxiety disorders. Of note, most studies that compared clinical or biological correlates of ADS relative to comorbid anxiety disorders in MDD grouped several different anxiety disorders together (e.g., GAD, SAD, PD), thereby increasing heterogeneity in this group. Thus, future studies in larger samples might investigate distinct comorbid anxiety disorders in MDD separately. Alternatively, it may be promising to distinguish between psychological, behavioral, and physiological symptoms of anxiety in neurobiological and clinical research (Taschereau‐Dumouchel et al., 2022). Of relevance, MDD/ADS+ scored higher on several other anxiety measures and depressive symptom severity compared to the MDD/ADS− group (see also Gaspersz et al., 2017b), which may pose alternative explanatory factors for the current findings. Although the abovementioned group differences in amygdala responses to emotional faces remained significant after correction for depression severity, the anxious distress group is known to be a more severe and chronic group of depressed patients relative to non‐anxious depressed patients (e.g., Fava et al., 2004; Gaspersz et al., 2017b; Hasin et al., 2018), making it difficult to truly disentangle different dimensions of anxiety and depression severity levels.

### 4.4 Clinical implications

The current study further builds on the work suggesting the DSM‐5 ADS is a clinically relevant aspect of depression. With a simple 5‐item questionnaire, it is relatively easy to administer in the clinic. A better understanding of the neurobiological underpinnings of anxious depression is highly relevant, especially considering worse treatment and trajectory predictions observed in anxious depression. Neuro‐informed interventions based on biotypes or specifically targeting affected neural circuitry may improve future treatment options for mood and anxiety patients (Williams, 2016). Based on the current findings, amygdala and striatal hyperreactivity may be a promising target specifically in anxious depression, though we must emphasize the cross‐sectional nature of the current work. Interestingly, a previous study partially overlapping with the current sample showed that baseline neural responses to the emotional faces task were predictive of MDD chronicity 2 years later (Schmaal et al., 2015). Considering that presence of ADS also showed strong predictive value of clinical trajectory within the current and other samples (Gaspersz et al.,, 2017b; Rosellini et al., 2018), combining ADS and amygdala reactivity may be a promising combination for prediction. Although the current sample was not large enough to perform ADS by prospective chronicity analyses, investigating ADS and limbic/striatal hyperreactivity in relation to MDD chronicity would be of interest for future naturalistic longitudinal neuroimaging studies, as well as clinical intervention studies. For example, neurofeedback interventions may be successful in amygdala downregulation (Nicholson et al., 2018; Young et al., 2018). Pharmacological and psychotherapy interventions may also dampen amygdala reactivity, both selective serotonin reuptake inhibitors and cognitive behavioral therapy attenuated amygdala responses to emotional faces in depression and anxiety patients, and the level of amygdala attenuation was associated with reduction of anxiety but not depressive symptoms (Gorka et al., 2019). Future studies may further assess whether targeting neurobiological hallmarks of anxious depression, such as limbic and striatal hyperreactivity, is successful in improving clinical outcomes.

## 5 CONCLUSION

Current findings suggest that amygdala and striato‐limbic hyperactivity to emotional faces may be a neurobiological hallmark specific to MDD with ADS, relative to MDD without anxious distress. The ADS was not convincingly associated with resting‐state functional connectivity patterns in salience and basal ganglia networks, and the subthreshold observed SN hyperconnectivity may have been related to depression diagnosis rather than anxious distress. The DSM‐5 ADS is a clinically relevant, easy to administer concept, and the current findings shed a light on the possible neurobiological mechanisms underlying anxious distress in MDD and its clinical presentation. This furthermore underlines the importance to account for heterogeneity in depression research and treatment. Though replication studies are necessary, this study provides a better understanding of the neurobiology of anxious distress, and may in the future help to improve and personalize treatment options for individuals with anxious depression.

## CONFLICT OF INTEREST

The authors declare no conflicts of interest.

## Supporting information

Supporting information.

## Data Availability

The data that support the findings of this study are available on request from the NESDA cohort study. The data sharing policy of NESDA and information on how to request for access to study data is available at https://www.nesda.nl/nesda-english/
