# Supplementary material for: Neural correlates of anxious distress in depression: A neuroimaging study of reactivity to emotional faces and resting‐state functional connectivity
Source: Depress Anxiety. 2022 May 10;39(7):573–85. doi: 10.1002/da.23264 (PMC9543619; doi:10.1002/da.23264)
Supplement: Supplementary file 1 — Supporting information. [file DA-39-573-s001.docx]

**SUPPLEMENTARY MATERIAL**

Neural correlates of anxious distress in depression, Nawijn et al

**A. SUPPLEMENTARY METHODS**

# **A1. Emotional faces paradigm**

Color photographs of angry, fearful, sad, happy and neutral facial expressions from the Karolinska Directed Emotional Faces System (KDEF, Lundqvist, Flykt & Öhman, 1998) were presented to all participants, in addition to a control condition consisting of scrambled faces. Twenty-four stimuli were selected for each of five facial expressions, comprising 12 female and 12 male faces. The control condition (scrambled faces) was presented 80 times. An event-related design was used involving a pseudo-random presentation of a total of 200 stimuli against a black background. Each photograph was shown on the screen for 2.5 s, with an interstimulus (black screen) interval varying between 0.5 and 1.5 s. Participants were instructed to indicate each face’s gender by pressing one of two buttons with the index finger of the left or right hand on two button boxes. During the presentation of scrambled faces, participants had to press left or right buttons in conformity with the instruction presented on the screen. The emotional faces task-scan lasted 7 minutes and 40 seconds. Resting state scan and emotional faces paradigm were administered as part of a fixed imaging protocol (protocol sequence: planning task, word encoding task, T1-weighted scan, word recognition task, emotional faces task, resting state scan).

*Reference:*

- *Lundqvist D, Flykt A, Ohmann A (1998). The Karolinska Directed Emotional Faces (KDEF). Karolinska Institute: Stockholm.*

# **A2. Imaging acquisition and preprocessing**

A T1-weighted anatomical MRI was acquired for each subject (repetition time (TR)=9 ms, echo time (TE)=3.5 ms, flip angle=8º, Field of view (FOV) matrix size=256×256, voxel size=1×1×1 mm). For the emotional faces task, a T2*-weighted gradient-echo echoplanar imaging (EPI) was used (Amsterdam and Leiden: 200 whole-brain volumes, TR=2300 ms, TE=30 ms, flip angle=80º, 35 transverse slices, no slice gap, FOV=220×220 mm, voxel size=2.3×2.3 mm, slice thickness 3 mm; Groningen: similar except TE=28 ms, 39 axial slices, voxel size=3.45×3.45 mm). Resting state fMRI data were acquired using T2*-weighted gradient-echo EPI (Amsterdam and Leiden: 200 whole-brain volumes, TR=2300 ms, TE=30 ms, flip angle=80º, 35 axial slices, no slice gap, FOV=220×220 mm; voxel size=2.3×2.3 mm, slice thickness=3 mm; Groningen similar except for: TE=28 ms, 39 axial slices, voxel size=3.45×3.45 mm).

Preprocessing of emotional faces and resting state data was conducted using FSL 5.0.8 and included artefact removal using FSL FIX (Salimi-Khorshidi et al., 2014), motion correction (realignment), grand mean scaling, spatial smoothing with 6mm Gaussian kernel, motion artefact removal using ICA-AROMA – which includes but is not limited to removing motion-related artefacts explicitly correlating with the realignment parameters - (Pruim, Mennes, Buitelaar, & Beckmann, 2015; Pruim, Mennes, van Rooij, et al., 2015) and high-pass filtering (emotional faces cut-off 80 seconds; resting state cut-off 100 seconds). For the resting state scan additional nuisance signal regression of white matter and cerebrospinal fluid was performed. First-level statistical parametric maps were created using FSL FEAT for the emotional faces task (for contrasts see statistical analyses). The first-level statistical parametric maps and resting-state scans were registered to Montreal Neurological Institute (MNI) space using registration matrices obtained from first co-registration of functional image to T1 image using boundary-based registration tool and registering the T1 images to MNI template brain. Participants were excluded if movement was >|2.5| mm / |0.4| rad, or if functional images were of insufficient quality. Quality control was performed by two researchers. Participants were excluded if both researchers scored their quality as insufficient. Inconsistencies were discussed until consensus was reached.

*References:*

- *Salimi-Khorshidi, G., Douaud, G., Beckmann, C. F., Glasser, M. F., Griffanti, L., & Smith, S. M. (2014). Automatic denoising of functional MRI data: Combining independent component analysis and hierarchical fusion of classifiers. NeuroImage, 90, 449–468.*
- *Pruim, R. H. R., Mennes, M., van Rooij, D., Llera, A., Buitelaar, J. K., & Beckmann, C. F. (2015). ICA-AROMA: A robust ICA-based strategy for removing motion artifacts from fMRI data. NeuroImage, 112, 267–277.*
- *Pruim, R. H. R., Mennes, M., Buitelaar, J. K., & Beckmann, C. F. (2015). Evaluation of ICA-AROMA and alternative strategies for motion artifact removal in resting state fMRI. NeuroImage, 112, 278–287.*

**B. SUPPLEMENTARY RESULTS**

**EMOTIONAL FACES**

# **Supplementary Table S1.** Emotional faces task activation

| **Clusters of activation** | **Voxels** | **Peak-coordinates** | | | **Z value** | **p-value** |
| --- | --- | --- | --- | --- | --- | --- |
|  |  | **x** | **y** | **z** |  |  |
| Right amygdala, hippocampus, precentral gyrus/IFG, frontal pole | 4031 | 18 | -6 | -16 | 10.40 | p<0.001 |
| Right fusiform cortex, occipital pole | 2097 | 42 | -48 | -20 | 11.10 | p<0.001 |
| Left amygdala, hippocampus, temporal pole | 1399 | -18 | -6 | -14 | 10.70 | p<0.001 |
| Left fusiform cortex, occipital pole | 847 | -38 | -50 | -20 | 9.89 | p<0.001 |

*Task effect of emotional faces task, contrast of emotional faces > scrambled faces across participants. Threshold Z=2.3.*

#
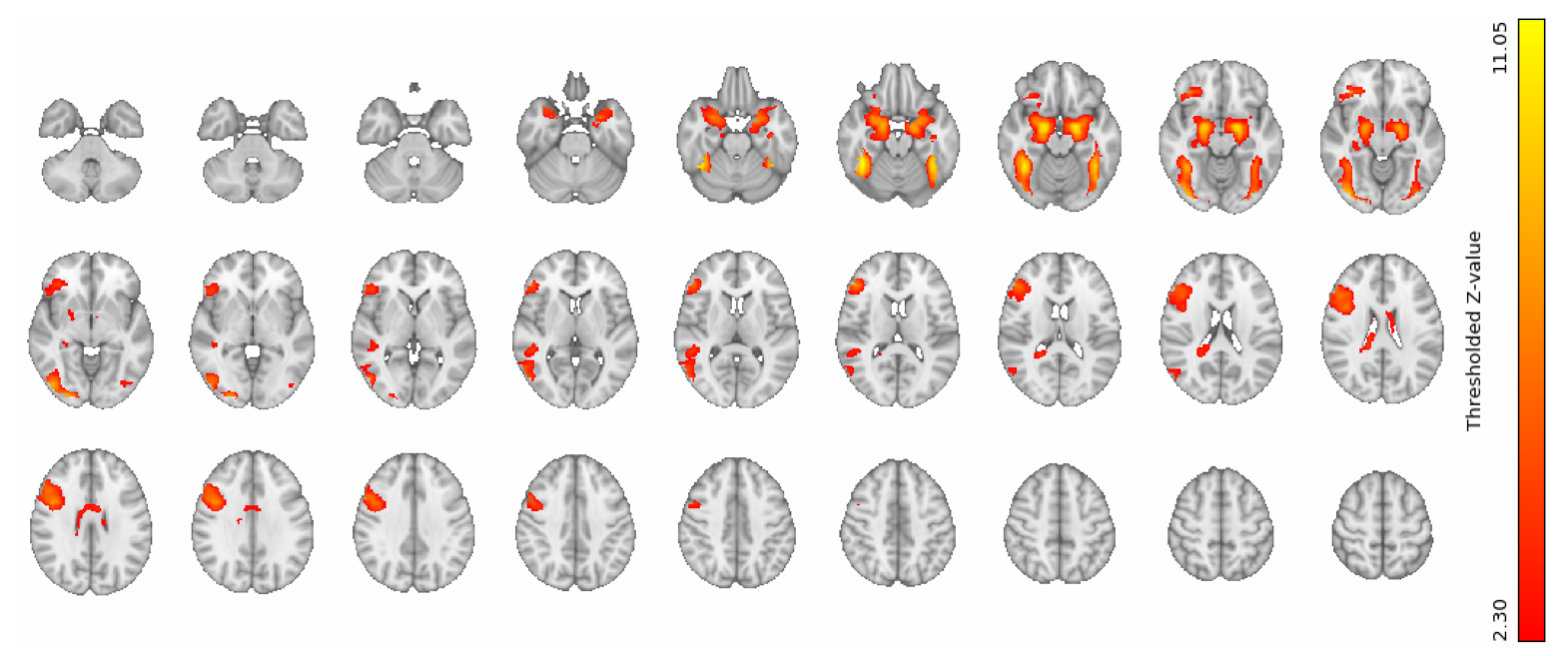


# **Supplementary Figure S1.** Emotional faces task activation.

*Task activation of emotional faces task, contrast of emotional faces > scrambled faces across participants, showing significant task activation in the bilateral amygdala and hippocampus, fusiform cortex and right inferior frontal gyrus. Threshold Z=2.3 for activation clusters, overlaid on MNI template.*

**Supplementary Table S2.** Post-hoc analyses of amygdala reactivity to happy, angry, sad and fearful faces in MDD/ADS+ vs MDD/ADS-.

| **Clusters of activation** | **Voxels** | **Peak-coordinates** | | | **Z value** | **p-value** |
| --- | --- | --- | --- | --- | --- | --- |
|  |  | **x** | **y** | **z** |  |  |
| **Amygdala ROI analyses** |  |  |  |  |  |  |
| *Emotional faces (happy, angry, sad, fearful)* |  |  |  |  |  |  |
| Left amygdala | 167 | -14 | 2 | -14 | 4.02 | 0.015* |
| Right amygdala |  |  |  |  |  | NA |
| *Happy faces* |  |  |  |  |  |  |
| Left amygdala | 57 | -14 | -2 | -14 | 3.61 | 0.114 |
| Right amygdala |  |  |  |  |  | NA |
| *Angry faces* |  |  |  |  |  |  |
| Left amygdala | 114 | -14 | -2 | -14 | 3.11 | 0.038* |
| Right amygdala |  |  |  |  |  | NA |
| *Sad faces* |  |  |  |  |  |  |
| Left amygdala | 77 | -14 | -2 | -16 | 3.60 | 0.076 |
| Right amygdala | 77 | 20 | -8 | -18 | 3.43 | 0.076 |
| *Fearful faces* |  |  |  |  |  |  |
| Left amygdala | 95 | -16 | -8 | -22 | 3.26 | 0.052 |
| Right amygdala | 49 | 24 | -4 | -8 | 3.27 | 0.136 |

*Post-hoc analyses of group differences for contrast MDD/ADS+ > MDD/ADS- in amygdala reactivity (bilateral amygdala ROI) on emotional faces task for all emotional faces together and per emotional expression separately (contrasts emotional faces > scrambled faces). * = p<0.05, NA = not applicable, no group differences observed.*

# **B1. Post-hoc sensitivity analyses: Amygdala and putamen reactivity to emotional faces in MDD with anxious distress**

To investigate if the differences between MDD/ADS+ and MDD/ADS- groups in amygdala (MNI-coordinates *x,y,z* = -14, -2, -14, *Z*=4.02, *k*=167, *p*=0.015) and putamen reactivity (MNI-coordinates *x,y,z* = -28, 6, 2, *Z*=4.28, *k*=372, *p*=0.052) were dependent on comorbid anxiety disorders or depression severity, post-hoc sensitivity analyses were run on mean percent signal change values extracted from the group-comparison left amygdala and putamen clusters.

ANOVA’s and independent-sample t-tests in SPSS on extracted amygdala mean percent signal change suggested that ADS-amygdala effects were not driven by comorbid anxiety: Differences in amygdala reactivity between MDD/ADS+ and MDD/ADS- were still significant when correcting for comorbid anxiety (*F*(1,69)=15.607, *p*<0.001) and when excluding MDD participants with comorbid anxiety (*t*(36)=-2.656, *p*=0.012, n=34 excluded). Also, MDD/ANX+ did not differ from MDD/ANX- in left amygdala cluster reactivity (*t*(70)=-1.073, *p*=0.287) nor from controls (*t*(88)=-0.697, *p*=0.488). Furthermore, MDD/ADS group differences were still significant when additionally correcting for depression severity (IDS scores, *F*(1,69)=12.816, *p*=0.001).

Similar results were obtained for MDD/ADS+ putamen cluster hyperreactivity. ANOVA’s and independent-sample t-tests in SPSS on extracted putamen mean percent signal change suggested that ADS-putamen effect were not driven by comorbid anxiety: Differences in putamen reactivity between MDD/ADS+ and MDD/ADS- were still significant when correcting for comorbid anxiety (*F*(1,69)=16.383, *p*<.001) and when excluding MDD participants with comorbid anxiety (*t*(36)=-2.261, *p*=0.030; n=34 excluded), and MDD/ANX+ did not differ from MDD/ANX- in left putamen cluster reactivity (*t*(70)=0.391, *p*=0.697) nor from controls (*t*(88)=0.672, *p*=0.503). Furthermore, MDD/ADS group differences were still significant when additionally correcting for depression severity (i.e. IDS scores; *F*(1,69)=18.055, *p*<.001).


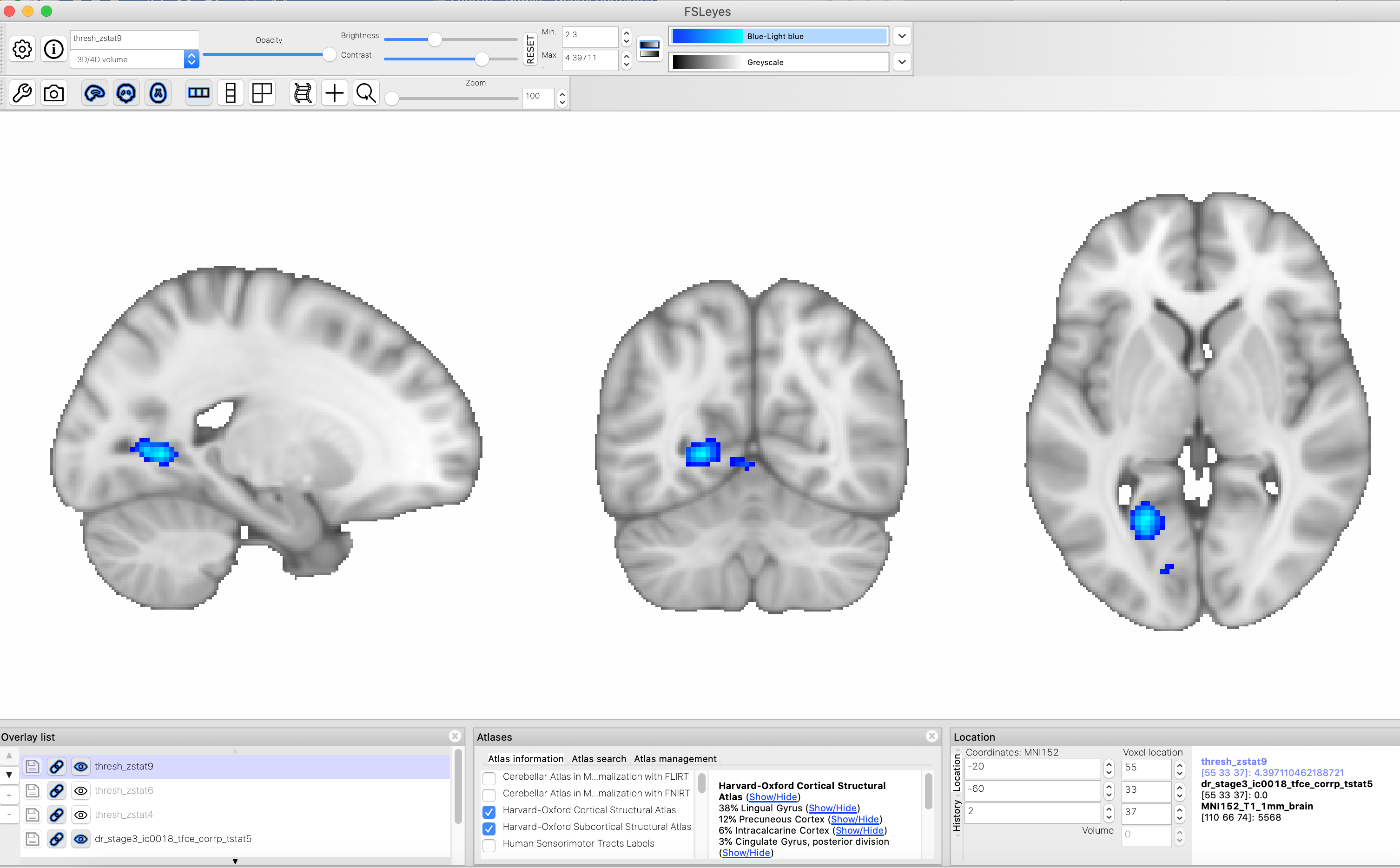


**Supplementary Figure S2.** Decreased left lingual gyrus and precuneus responses to emotional faces in MDD compared to controls.

*MDD patients show decreased left lingual gyrus / precuneus responses to emotional faces relative to healthy controls, when performing whole brain analysis (MNI-coordinates xyz -20, -60, 2, Z=4.40, k=405, p=0.035). Heatmaps correspond to Z-values, threshold Z≥2.30, and are overlaid on an MNI standard brain template, right hemisphere in image corresponds to right hemisphere of the brain.*

**RESTING STATE ANALYSES**


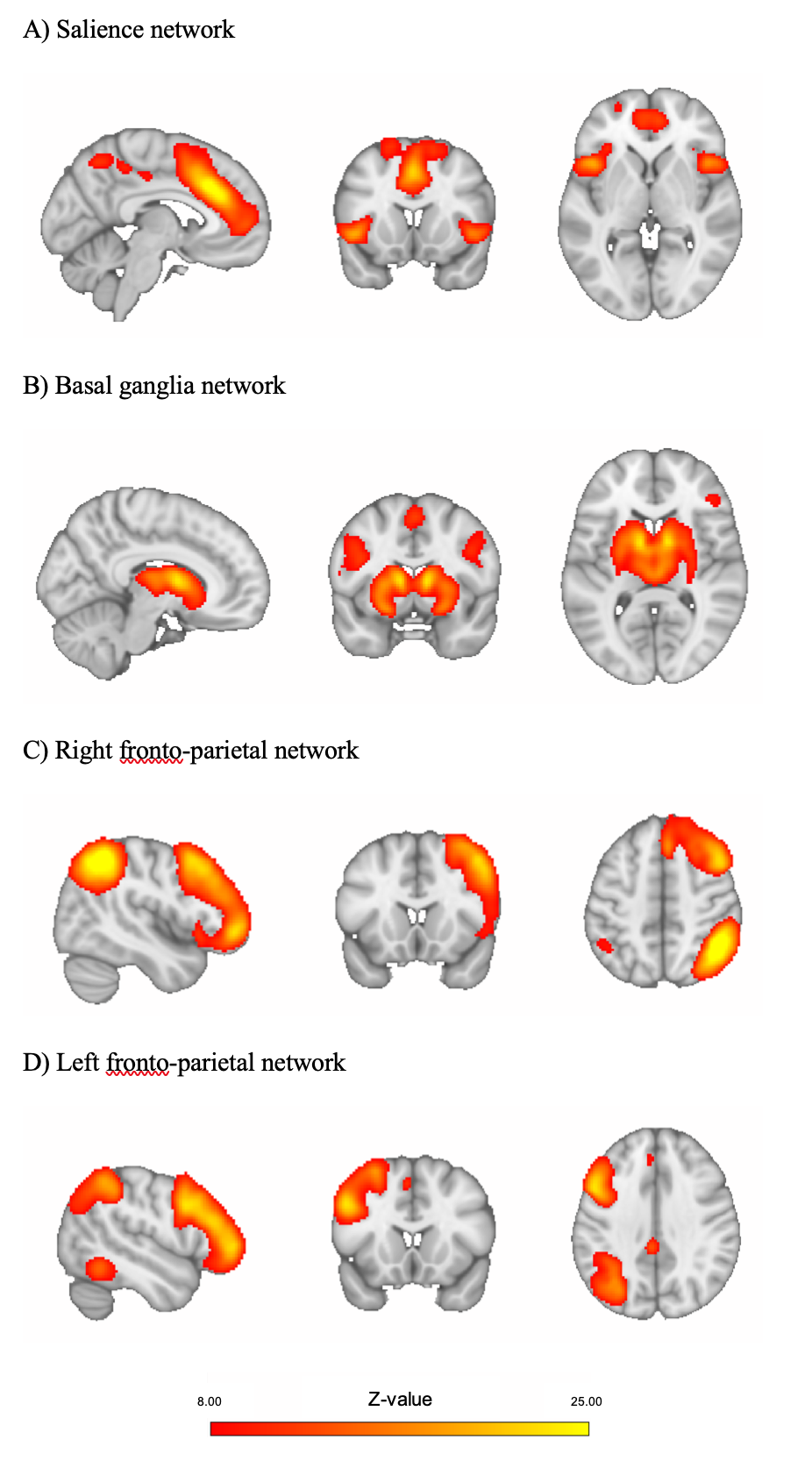


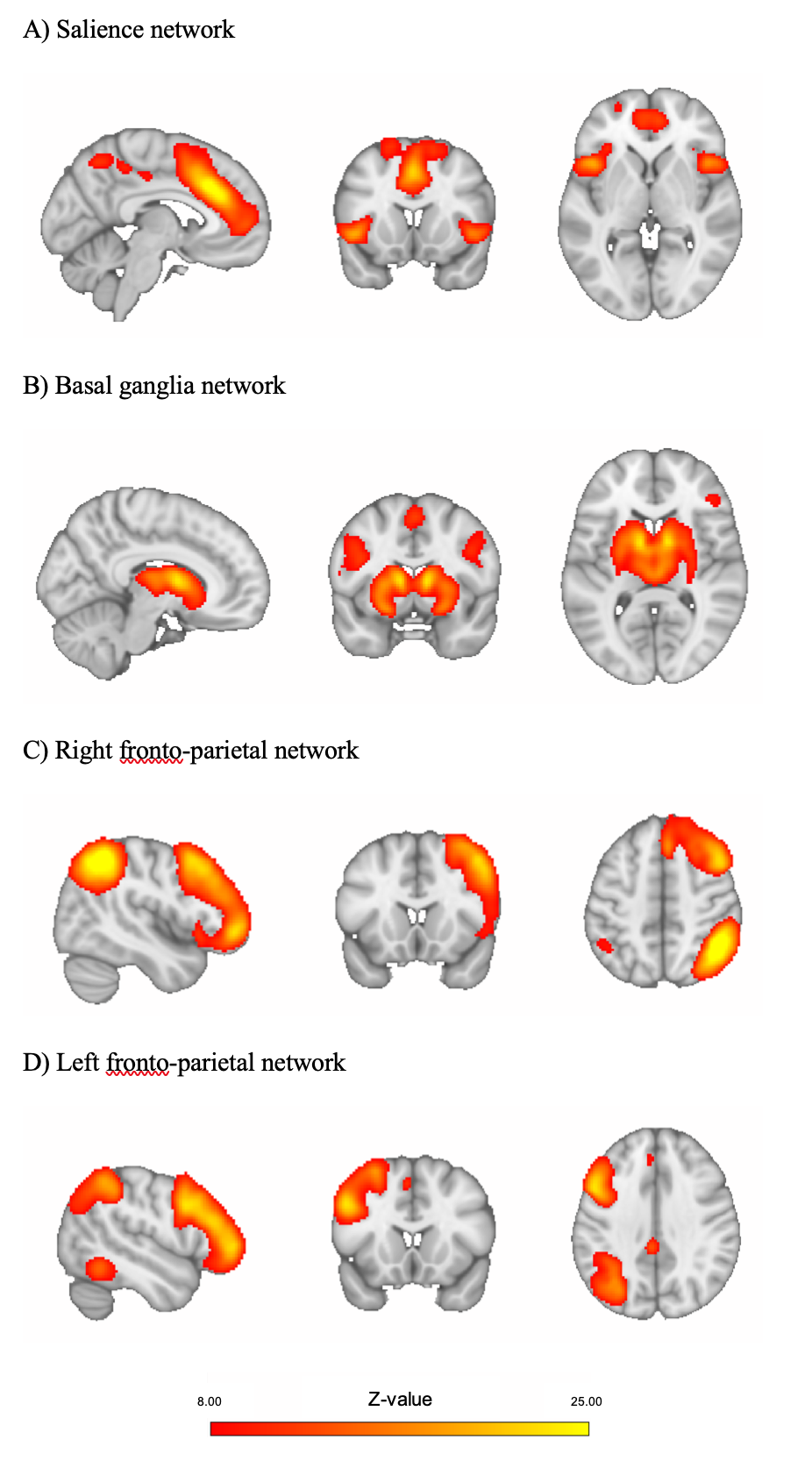


**Supplementary Figure S3.** Salience and basal ganglia intrinsic functional connectivity networks of interest**.**

*Resting state network independent components selected for further analyses, based on one-sample independent component analysis (i.e. concatenating datasets of all participants).* ***A: Salience network*** *(xyz -4, 12, 1), including bilateral anterior insula, dorsal anterior cingulate cortex. Correlation with intrinsic connectivity netwerk (ICN) 4 from Laird et al. (2011) was r=0.31.* ***B: Basal ganglia network*** *(xyz, 10, 4, 9), including bilateral thalamus, caudate, putamen, amygdala, hippocampus, dmPFC [paracingulate gyrus/suppl motor cortex], dlPFC [precentral gyrus, IFG]. Correlation with ICN 3 from Laird et al. (2011) was r=0.50. Heatmaps corresponds to Z-values, threshold Z≥8.00, and are overlaid on an MNI standard brain template, right hemisphere in image corresponds to right hemisphere of the brain.*

**
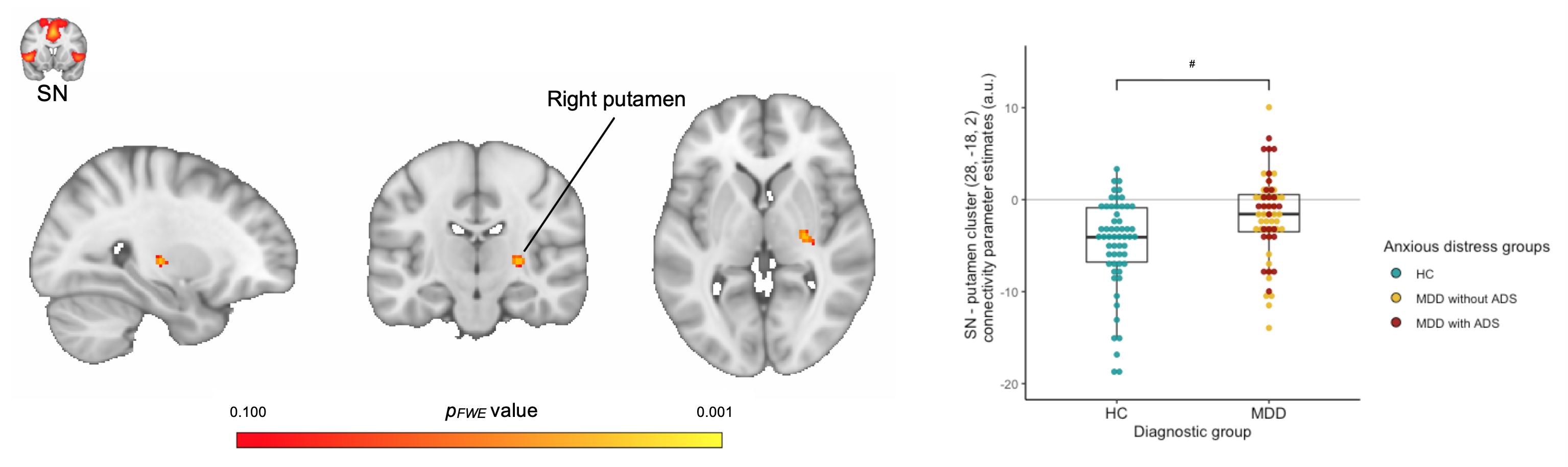
**

**Supplementary Figure S4.** Salience functional connectivity in MDD participants relative to controls.

*Subthreshold stronger salience network (SN)-right putamen functional connectivity in MDD participants relative to controls (MDD_ALL_>HC, xyz 28, -18, 2, p=0.026, p_FDR_=0.052), image top left showing anterior view of the SN. Dot-plots and boxplots illustrate the group differences, based on extracted mean parameter estimates from a 5mm sphere around the peak voxel (arbitrary units). Heatmaps corresponds to p-values and are overlaid on an MNI standard brain template. Right hemisphere in image corresponds to right hemisphere of the brain. Abbreviations: SN, salience network; a.u., arbitrary units; HC, healthy control; MDD_ALL_, participants with major depressive disorder; ADS, DSM-5 anxious distress specifier. # = p<0.050 based on whole brain neuroimaging group-comparisons, but not significant after FDR-correction.*
